# No Robust Effect of Distributed Practice on the Short- and Long-Term Retention of Mathematical Procedures

**DOI:** 10.3389/fpsyg.2020.00811

**Published:** 2020-04-29

**Authors:** Mirjam Ebersbach, Katharina Barzagar Nazari

**Affiliations:** Division of Developmental Psychology, Department of Psychology, University of Kassel, Kassel, Germany

**Keywords:** desirable difficulties, distributed practice, spacing, lag effect, mathematics learning, procedural knowledge

## Abstract

We investigated the effect of distributed practice and more specifically the “lag effect” concerning the retention of mathematical procedures. The lag effect implies that longer retention intervals benefit from longer inter-study intervals (ISIs). University students (*N* = 235) first learned how to solve permutation tasks and then practiced this procedure with an ISI of zero (i.e., massed), one, or 11 days. The final test took place after one or five weeks. All conditions were manipulated between-subjects. Contrary to our expectations, the analyses revealed no effect of distributed practice and therewith also no lag effect, even though the sample size was sufficiently large. The only significant effect was that test performance was poorer after 5 weeks than after 1 week. In view of the present results and those of other studies, we assume that distributed practice works differently for declarative and procedural knowledge, with less robust of even absent effects when procedural skills are practiced with ISIs compared to massed practice.

## Introduction

One central aim of research in cognitive and educational psychology is to identify mechanisms that improve short- and long-term retention. So called *desirable difficulties* refer to mechanisms that make the learning process subjectively harder but contribute to a longer maintenance of knowledge (Bjork, 1994). Distributing the total learning time across several sessions instead of learning the same contents in only one session in a massed fashion—also called *distributed practice* or *spacing*—is one of these desirable difficulties. In general, distributed practice yielded robust effects in experimental settings (e.g., Cepeda et al., 2006; Carpenter et al., 2012) and can therefore be considered as a powerful learning tool (Gerbier and Toppino, 2015; Kang, 2016).

Several theoretical accounts try to explain the effect of distributed practice (for overviews see Küpper-Tetzel, 2014; Toppino and Gerbier, 2014). It is, for instance, assumed that distributed practice can impede *deficient processing* because it prevents the metacognitive impression in learners that the to be learned items are already familiar and therewith consolidated (i.e., feeling of knowing). Accordingly, a higher attentive maintenance is devoted to the processing of spaced items compared to massed items (Craik and Lockhart, 1972; Hintzman, 1974; Crowder, 1976; Challis, 1993). Another account assumes that *working memory depletion* occurs in massed practice but not in distributed practice due to the intervals between the practice sessions (Chen et al., 2018). Furthermore, distributed practice fosters *study-phase retrieval processes* because learners have to remember what they have practiced in the session(s) before due to the intervals between practice sessions (Toppino et al., 2018). Such retrieval practice fosters the consolidation of knowledge by increasing the storage strength of the learned material (Bjork, 1975; Thios and D’Agostino, 1976; Bjork and Bjork, 1992, 2006; Braun and Rubin, 1998; Smith and Scarf, 2017). Distributed practice can also enhance the *variability of the learning contexts* (i.e., mental, physical, and experimental contexts). This variability facilitates recall because it provides more potential retrieval cues (Estes, 1955; Tulving and Thomson, 1973; Glenberg, 1979; Raaijmakers, 2003; Maddox, 2016). All of these mechanisms are not excluding each other and may work simultaneously.

Besides the general effect of distributed practice, some studies were concerned with the optimal length of the intervals between the single learning sessions (i.e., inter-study interval, ISI) to yielding maximal benefits of distributed practice for retention. Cepeda et al. (2008), using verbal material (i.e., true trivia facts), showed systematically that the optimal ISI depends on the timing of the final test (i.e., “lag effect”; Glenberg, 1976). Generally, longer retention intervals (RI) between the last learning session and a final test require longer ISI, although this relationship is considered to be non-monotonic (cf. Donovan and Radosevich, 1999; Janiszewski et al., 2003; Cepeda et al., 2009). Küpper-Tetzel and Erdfelder (2012) manipulated the ISI (none – which is equivalent to massed practice; 1 day; and 11 days) and RI (one; five weeks) systematically among adults who had to learn word pairs. Recall after 1 week was best when the ISI was 1 day (see also Cepeda et al., 2008), while recall after 5 weeks was best with an ISI of 11 days (see also Küpper-Tetzel et al., 2014a). Moreover, a contracting ISI (i.e., inter-study intervals becoming shorter between multiple learning sessions) yielded larger effects on the successful recall of word pairs within a period up to 1 week, compared to a constant or expanding ISI. In contrast, an expanding ISI yielded larger effects for a RI of 5 weeks (Küpper-Tetzel et al., 2014b). Moreover, an expanding ISI also yielded larger effects on the retention tested after 2 weeks, compared to a constant or contracting ISI, when participants had poor prior knowledge (i.e., a low-level initial training). No such effect was found for participants with prior knowledge (i.e., following a high-level initial training; Toppino et al., 2018). Thus, the length and timing of the ISI in distributed practice seem to be central factors for successful retention.

Most studies so far referring to distributed practice addressed declarative knowledge (e.g., facts, foreign vocabulary, names of objects or pictures, and understanding statistics concepts), with verbal recall as dependent variable (cf. Dempster, 1988; Cepeda et al., 2006; Carpenter et al., 2012; Kang, 2016). This is a clear shortcoming because knowledge acquisition in formal and informal learning contexts often includes procedural knowledge. For instance, in mathematics, it is central to be able to execute procedures fluently and accurately. However, much fewer studies considered the effect of distributed practice with regard to procedural knowledge.

In one of these studies, students watched consecutive, video-taped lessons on descriptive, and inferential statistics either massed on 1 day or distributed over 4 days (Smith and Rothkopf, 1984). In a test after 5 days, students’ factual knowledge (as assessed by “general recall,” “cued recall,” and “matching,” including for instance the naming three measures of central tendency) was better in the distributed compared to the massed condition. However, their procedural knowledge (as assessed by “problems,” that is, executing calculations) showed no such effect.

Rohrer and Taylor (2006, 2007) asked college students to learn and practice the solving of permutation tasks (i.e., identifying the number of unique orderings of a sequence of letters using a simple formula) in either a massed (i.e., two sessions successively on the same day) or distributed manner (i.e., two sessions with an ISI of 1 week). Students in the distributed condition performed better than participants in the massed condition on a final test after 1 week (Rohrer and Taylor, 2007) and after 4 weeks (Rohrer and Taylor, 2006)—but not after 1 week in the latter study.

Hopkins et al. (2016) spaced or massed quizzes in the context of a pre-calculus course for engineering students. The target skills addressed in the quizzes and the final exams involved declarative knowledge (e.g., understanding the definition of a logarithmic function) and procedural skills (e.g., solving quadratic equations by factoring and the zero product property). The spacing of quizzes referring to previously learned pre-calculus units across the whole duration of the course led to a better performance in the subsequent exams than massing the quizzes (for a similar study, see Lyle et al., 2019).

In another study with third-graders practiced addition problems over a period of 19 days in total (Schutte et al., 2015), either massed (4 min) or distributed (i.e., 2 min in the morning and 2 min in the afternoon, or four times for 1 min each, distributed over the whole day). Distributed practice outperformed massed practice in an immediate test and in a test after 10 days. However, actually all children practiced in a distributed manner across the period of 19 days, and a pure massed condition was not realized in this study.

Barzagar Nazari and Ebersbach (2019) directly compared distributed with massed practice concerning the learning of mathematical procedures among third and seventh graders in school. Pupils were first introduced to the procedure (i.e., semiformal multiplication or basic probability calculation). Thereafter, they practiced these procedures on 1 day for 45 min or on three consecutive days for 15 min each. The distributed practicing students outperformed the massed practicing students after 1 week and after 6 weeks, except for third graders, were the effect disappeared after 6 weeks. Chen et al. (2018) also revealed an effect of distributing practice across three consecutive days, including worked examples of adding fractions, on their task solving performance, tested at the fourth day.

Thus, even as there is some evidence that distributed practice might also enhance procedural skills in mathematics, the results are not always as straightforward (e.g., Smith and Rothkopf, 1984; Rohrer and Taylor, 2006; Barzagar Nazari and Ebersbach, 2019). One reason for the inconsistent findings might be the lag effect: The ISI might have been too short or too long for the particular RI realized in the respective studies. However, the lag effect has, so far, not been tested with regard to science-related procedural skills (e.g., mathematical procedures). Given that the acquisition and long-term maintenance of mathematical procedures is central for mathematics learning and that studies examining the effect of distributed practice are scarce in this regard, further research is needed.

The present study^1^ seizes this issue by examining the effect of distributed practice on the retention of mathematical procedures in a controlled experimental study with university students. We addressed the lag effect by manipulating the ISI as well as the RI systematically between-subjects, assessing short- and long-term effects. In line with the lag effect, we expected an interaction between ISI and RI. For a RI of 1 week, an ISI of 1 day should yield the largest effect, followed by an ISI of 11 days, and both ISIs should be better than no ISI (i.e., massed condition). For a RI of 5 weeks, in contrast, an ISI of 11 days should yield the largest effect, followed by an ISI of 1 day, and both ISIs should yield better results than no ISI (i.e., massed condition; cf. Cepeda et al., 2008).

Furthermore, we examined exploratorily whether individual learner characteristics moderate the effect of distributed practice. This issue has largely been neglected in previous research addressing desirable difficulties in learning. However, in order to provide learners and teachers with recommendations concerning optimal learning strategies, individual differences need to be addressed. We assumed that learners with a poorer *working memory capacity* might be over-challenged by distributed practice because they may stronger be affected by interferences that might occur between the practice sessions and, therewith, face more problems to maintain the primary task goals (Bui et al., 2013; but see Seabrook et al., 2005; Delaney et al., 2018). Furthermore, in line with deficient processing accounts (e.g., Craik and Lockhart, 1972), we expected participants with *difficulties to concentrate* on longer tasks to benefit especially from distributed practice because here, the duration of the single learning sessions is shorter and puts less demands on (longer-term) concentration. In addition, learners’ *performance avoidance goals*, *work avoidance*, and *effort motivation* were assessed as potential moderators of the effect of distributed practice. We hypothesized that performance avoidance reduces learners’ motivation to engage in the distributed practice condition as this condition requires more retrieval than the massed condition, and unsuccessful retrieval might be discouraging for this group of learners in particular. Furthermore, distributed practice is considered as a desirable difficulty in learning (Bjork, 1994), it is more effortful than cramming, and is therefore rarely used in the context of self-regulated learning (Barzagar Nazari and Ebersbach, 2018). Thus, learners scoring high in work avoidance and effort motivation might be less engaged in the distributed practice condition and, therefore, might benefit less or even not at all from the distribution. These moderator hypotheses were tested exploratorily with regard to the general effect of spacing; no specific moderator hypotheses were postulated concerning the interplay between different ISIs and RIs (see preregistration).

## Materials and Methods

### Design

The study followed a 3 (ISI: none vs. 1 day vs. 11 days) ×2 (RI: one vs. five weeks) between-subjects design. The dependent variable was the performance in the final test tapping procedural knowledge (i.e., solving permutation tasks; Rohrer and Taylor, 2006, 2007).

### Sample

The initial sample consisted of 273 students. Thirty-eight students had to be excluded because they had terminated the study before the end (*n* = 18), had taken part in a similar study before involving the same content (*n* = 12), or for other reasons (*n* = 8). The number of drop-outs in each condition was small and unsystematic, ranging between one to five participants. The final sample consisted of 235 students^2^ (mean age: *M* = 24 years 5 months; 176 women, 59 men) of a wide range of study programs, being randomly assigned to each of the six experimental conditions (*n* = 38–40 per condition). Students took part with informed consent and could terminate the experiment at all times. They received 15 Euro when they completed all three sessions or part of the total sum if they dropped out beforehand.

### Material

Participants received a computer-based tutorial introducing how to calculate permutations, and worked the practice tasks and test tasks on the computer, too. The tasks were adopted from Rohrer and Taylor (2007; e.g.: “In how many unique ways can the letters *abbccc* be arranged?”; for an overview of all practice and test tasks, see section “Appendix A”). Individual characteristics were assessed via computerized questionnaires and tasks: Working memory capacity by the Corsi Block Task (Kessels et al., 2000) and the Digit Span Backwards test (Woods et al., 2011), learners’ ability to concentrate by the Sustained attention test CPT-AX (Rosvold et al., 1956), learners’ effort motivation by the LIST (Wild and Schiefele, 1994; Cronbachs alpha: α = 0.74), and learners’ performance avoidance goals and work avoidance by two scales of the SELLMO (Spinath et al., 2012; split-half reliability: *r* = 0.73–0.78), both adapted to mathematics learning.

### Procedure

The study was realized as computerized experiment in the laboratory. Students practiced and were tested in small groups up to four persons but the tasks were performed individually on the computer. Each student was randomly assigned to one of the six experimental groups comprising between 38 and 40 students (see section “Design”). Students’ prior knowledge on the learning subject (i.e., permutations) was not assessed because no systematic effects were expected due to the random assignment of participants to the experimental conditions. However, students’ performance in the first practice set served as a control variable in the analyses. In the first session, the individual characteristics of all students were assessed^3^, and students in the distributed condition also received a tutorial explaining the formula to compute permutations and completed the first four of eight practice tasks. In the second session (exactly 1 or 11 days later), students in the distributed condition worked the remaining four practice tasks, whereas students in the massed condition received the tutorial but now and thereafter completed all eight practice tasks in this second session. The practice tasks had to be worked without the tutorial, but students were provided with the full solution path of the first and second task as well as of the fifth and sixth task after they had completed the tasks. This type of incomplete feedback was chosen to save time and because it was assumed that presenting only half of the solution paths would be sufficient to support students’ comprehension of permutation. In the third session (exactly one or five weeks later), the unannounced final test was administered that was introduced as further practice session, including five permutation tasks (each with a time limit of 45 s). Each correct solution in the practice and test trials was scored with one point (i.e., max. 4 points in each of the two the practice sets, max. 5 points in the test). Students were informed at the beginning of the study about the schedule of the sessions but not about what would happen exactly in the sessions to prevent them from additional practicing. After the test, students were informed about the aim of this study and that the final session included a performance test.

## Results

Table 1 shows the mean performance score in each practice set (max. 4) and the mean score for the first task of practice set 2 (max. 1) as indicator of study-phase retrieval, separately for each condition. An exploratory analysis was conducted to test whether the performance between the first and second practice set changed. A repeated-measures ANOVA with practice set as within-subjects variable and ISI as between-subjects variable revealed no main effects of practice set and ISI, *p*s > 0.13, but an interaction of the two variables, *F*(2, 232) = 3.88, *p* = 0.022, and η*_*p*_*^2^ = 0.03. *Post hoc* tests showed that the practice performance in groups who practiced with an ISI of 11 days declined between the first and second practice set, *t*(77) = 2.49, *p* = 0.045 (Bonferroni-Holm corrected), whereas practice performance in the other groups did not decline, *p* > 0.50. Furthermore, to check for performance differences between the study and test conditions already in the first practice set, another exploratory ANOVA was computed with ISI and RI as independent variables. This analysis yielded no significant differences between the conditions, *p*s > 0.36.

**TABLE 1:** Mean performance in the practice sets 1 and 2, and in the first task of practice set 1 only, separately for each ISI and RI.

		**Inter-study interval**		
**Retention interval**	**Practice set**	**ISI 0**	**ISI 1**	**ISI 11**
RI 7	1	1.95 (1.69)	1.43 (1.50)	1.85 (1.59)
	2	2.18 (1.30)	1.45 (1.41)	1.43 (1.15)
	1st task of set 2	0.68 (0.47)	0.43 (0.50)	0.40 (0.50)
RI 35	1	1.79 (1.56)	1.82 (1.57)	1.55 (1.61)
	2	1.87 (1.34)	1.89 (1.43)	1.32 (1.23)
	1st task of set 2	0.62 (0.49)	0.61 (0.50)	0.34 (0.48)

*Mean scores (max. 4) of the performance in each practice set and in the first task of the second practice set (max. 1); standard deviations in parentheses.*

**FIGURE 1:**
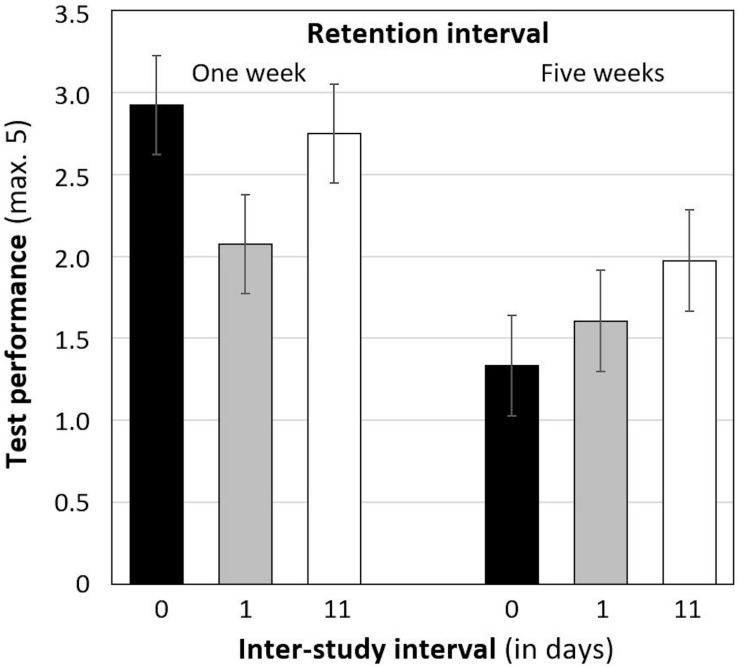
Mean test performance after a retention interval of 1 week (left) and 5 weeks (right), separately for each inter-study interval (standard errors in parentheses). *Note*. Per condition: *n* = 38 to 40 students.

Descriptive statistics of the test performance are shown in Figure 1. As outlined in the introduction, we expected an interaction between ISI and RI, as indicated by the best performance in a test after 1 week when students practiced with an ISI of 1 day, followed by an ISI of 11 days, and by massed practice. In contrast, performance in a test after 5 weeks was expected to be best when students practiced with an ISI of 11 days, followed by an ISI of 1 day, and by massed practice.

To test our hypotheses, a multiple linear regression was computed with ISI and RI as well as their interaction terms as predictors for the test performance. Because the normal distribution of the residuals could not be assumed (Shapiro-Wilk test: *p*s < 0.001), a robust multiple regression was computed based on bootstrapping. The regression model was significant, *F*(5, 229) = 4.21, *p* = 0.001, and *R*_adj_^2^ = 0.06. As shown in Table 2, the performance in a test after 5 weeks was in general poorer (*M* = 1.63, *SD* = 1.82) than in a test after 1 week (*M* = 2.53, *SD* = 2.01). However, contrary to our expectations, there was no main effect of ISI nor an interaction between RI and ISI.

**TABLE 2:** Results of a robust multiple regression testing main effects of ISI and RI and interactions.

**Predictor**	***B***	***SE***	***t***	**β**	***p***
ISI_A	–0.02	0.18	–0.11	–0.01	0.909
ISI_B	0.26	0.15	1.71	0.11	0.103
RI	–0.47	0.13	–3.79	–0.24	0.001**
RI × ISI_A	0.32	0.18	1.84	0.12	0.078
RI × ISI_B	–0.08	0.15	–0.50	–0.03	0.633

Based on 1000 bootstrap samples. ISI: inter-study interval; RI: retention interval. ISI_A: distributed practice with an ISI of 1 or 11 day compared to massed practice (ISI 0) as reference category; ISI_B: ISI 11 compared to ISI 1 as reference category; RI: RI 35 compared to RI 7 as reference category. RI × ISI_A and RI × ISI_B are the interaction terms. **p < 0.01.

### Exploratory Analyses

The sample size was not sufficient for computing moderator analyses to uncover potential moderating effects of the learners’ characteristics (for descriptive statistics, see Table 3) on the effect of distributed practice. These effects were therefore assessed exploratorily by means of conditional inference tree models (CIT, Hothorn et al., 2006, 2015). CIT models are—compared to more classical analyses, like multiple regressions—a flexible tool to uncover linear and non-linear associations between a dependent variable and multiple independent variables as well as interactions between independent variables. The variables can have different scales of measurement. Furthermore, CIT models facilitate the interpretation of complex regression problems by means of the visualization of the fitted decision trees (Zeileis et al., 2008). CIT models are based on recursive binary partitioning and first test globally whether the null hypothesis (i.e., that the dependent variable is independent from all tested independent variables) can be rejected. If this is the case, the independent variable with the strongest relationship to the dependent variable is chosen to divide the sample, based on two (or more) categories of the independent variable, into subgroups that maximally differ from each other with regard to the dependent variable. This process is iterated until the null hypothesis cannot be rejected anymore. A potential moderating effect of the learners’ characteristics would be reflected in such a pattern that the whole sample would be divided for instance in learners with lower and higher working memory capacity, and that only in the group of learners with higher working memory, an advantage of distributed practice would emerge, as mirrored in an additional splitting of this subgroup by means of the practice conditions. Given the exploratory character of these analyses, the results should be treated with caution but may provide interesting hints for future research. However, the analyses revealed none of the expected interaction effects (for a complete information on the results of the CIT analyses, see section “Appendix B”).

**TABLE 3:** Descriptive statistics of the learner characteristics per condition.

	**Condition**					
**Learner characteristics**	**ISI 0_RI 7**	**ISI 0_RI 35**	**ISI 1_RI 7**	**ISI 1_RI 35**	**ISI 11_RI 7**	**ISI 11_RI 35**
Working memory (Corsi Block)	6.25 (0.98)	6.08 (1.13)	6.00 (1.28)	6.21 (0.99)	6.18 (1.36)	5.79 (0.78)
Working memory (Digit Span)	6.90 (1.17)	6.77 (1.75)	6.30 (1.22)	6.92 (1.58)	6.90 (1.55)	6.61 (1.46)
Concentration	3.42 (1.10)	3.76 (0.84)	3.35 (1.06)	3.27 (0.93)	3.55 (1.04)	3.40 (0.92)
Effort motivation	4.20 (0.94)	4.12 (0.61)	4.34 (0.81)	4.34 (0.67)	4.31 (0.69)	4.31 (0.67)
Performance avoidance	2.41 (0.89)	2.52 (0.87)	2.46 (0.91)	2.41 (0.87)	2.58 (0.99)	2.42 (0.92)
Work avoidance	2.16 (0.88)	2.19 (0.67)	4.38 (2.38)	2.12 (0.62)	2.00 (0.71)	2.10 (0.73)

*Mean scores; standard deviations in parentheses.*

## Discussion

We investigated the effect of distributed practice on the retention of procedural knowledge in mathematics. More specifically, we aimed at examining the lag effect systematically to figure out whether learners tested after a shorter RI would benefit more from a shorter interval between the practice sessions ISI, whereas learners tested after a longer RI would benefit more from a longer ISI. Based on previous research investigating the lag effect with regard to verbal material, we expected a shorter ISI of 1 day to be optimal for a RI of 1 week, and a longer ISI of 11 days to be optimal for a RI of 5 weeks (e.g., Cepeda et al., 2009; Küpper-Tetzel et al., 2014a). In addition, in line with the general effect of distributed practice (e.g., Cepeda et al., 2006), a lag between the practice sessions (i.e., ISI > 0, distributed conditions) was expected to result in a better test performance than no lag (i.e., ISI = 0; massed condition). However, these expectations were not confirmed in our experiment: There was no main effect of ISI, no interaction between ISI and RI, only a main effect of RI: Learners performed poorer in a test after 5 weeks than in a test after 1 week, independently of the ISI.

Our findings are consistent with results of other studies that failed to demonstrate a robust effect of distributed practice with regard to mathematics. Rohrer and Taylor (2006), for instance, found no benefit of distributed compared to massed practice for the retention of mathematical procedures (i.e., permutation tasks like in the present study) when learners were tested after 1 week. Similarly, Smith and Rothkopf (1984) did not find an effect of distributing statistics lessons over 4 days on the solving of mathematical problems in a test 5 days later, and third-graders showed no benefit from the distributed practice of mathematical skills in a test after 6 weeks (Barzagar Nazari and Ebersbach, 2019). Also concerning other rather procedural skills, such as second language syntax, there was no performance difference in a test after 1 week depending on whether practice was distributed with an ISI of 3 days or with an ISI of 14 days (Bird, 2010). Unfortunately, a pure massed condition was not realized in this study. Other studies also failed to reveal a benefit of distributed practice for foreign language acquisition, including vocabulary, grammar, listening and reading (e.g., Lapkin et al., 1998; Collins et al., 1999; Serrano and Munoz, 2007).

However, when the learning addressed the recall of verbal material, not the application of procedures, distributed practice did yield quite reliably positive effects, even in a test after 1 week (e.g., Cepeda et al., 2008, using trivia facts; Küpper-Tetzel and Erdfelder, 2012 and Küpper-Tetzel et al., 2014a, using word pairs or vocabulary; for a review see Cepeda et al., 2006).

One might thus assume that the effect of distributed practice works differently for procedural and declarative knowledge. While declarative (or conceptual) knowledge refers to the understanding of concepts and the ability to recall facts related to these concepts, procedural knowledge additionally involves the construction of schemata and automation processes (Anderson, 1982). The discrepancy between both knowledge types in mathematics becomes evident already when one considers the timing of the development of mathematical skills in children. Depending on the particular skill, procedural or declarative knowledge emerges earlier (see for instance Rittle-Johnson and Schneider, 2015; Rittle-Johnson et al., 2015, for reviews).

The assumption that distributed practice might affect declarative and procedural knowledge differently is confirmed by a meta-analysis of Donovan and Radosevich (1999) revealing that the effect of the distribution depends on the *complexity* of the learning content. More complex content, requiring a number of distinct behaviors or mental procedures, benefits less (or not) from the distribution of practice compared to less complex content. Mathematical procedures can be conceived as being more complex as they include not only recalling the underlying concept (e.g., the formula) but also the ability to apply this concept within a task solving procedure. Thus, working memory is demanded stronger when executing procedures than when simply recalling previously learned word lists (e.g., Ashcraft and Krause, 2007). This complexity might have contributed to the finding of no advantage of distributed practice on procedural knowledge.

This assumption is supported by the finding that a testing effect—that is, a benefit for retention when previously learned information is retrieved already in the learning phase—remains absent or is even reversed when problem solving skills were addressed instead of incoherent learning material (van Gog and Kester, 2012; van Gog et al., 2015; van Gog and Sweller, 2015). van Gog et al. (2015) assume that practicing a problem-solving procedure as long as the underlying schema or concept is not fully consolidated might yield no additional benefits for the learning outcome. Moreover, Ullman (2004) refers to *interferences between procedural memory and declarative memory* in that the retrieval of declarative knowledge can sometimes hinder the retrieval of procedural knowledge (an vice versa).

Finally, given that procedural skills often involve motor elements (i.e., the execution of a procedure), it seems worthwhile to consider the effect of distributed practice on motor skills. A meta-analysis by Lee and Genovese (1988) revealed overall a medium effect on the retention of motor procedures. However, discrete and continuous procedures have to be differentiated with regard to this effect: Only continuous motor procedures, defined by an arbitrary beginning and end (e.g., rotary pursuit), benefit from distributed practice compared to massed practice. *Discrete motor procedures*, defined by a fixed beginning and end (e.g., throwing a ball), in contrast, benefit more from massed practice (e.g., Lee and Genovese, 1989; Garcia et al., 2008; Panchuk et al., 2013). If one conceives solving a mathematical task as involving a discrete (motor) task, this aspect might have additionally contributed to the absent benefit of distributed practice for this type of tasks.

To sum up, due to the procedural nature of solving arithmetic tasks, the effect of distributed practice on mathematical skills might be less pronounced or even absent (but see Chen et al., 2018, or Lyle et al., 2019, for positive effects). Further research is necessary to examine the effect of distributed practice on procedural skills, also including procedures from different subjects and a more fine-grained grading of ISI and RI. In addition, especially with regard to procedural skills, one should differentiate between the effects of distributed practice on practice performance (skill acquisition) and test performance (retention). Our data showed a significant decrease of the practice performance between practice set 1 and 2 when the two sets were separated by an ISI of 11 days but not for the shorter ISIs. It would also be interesting to examine the effect of increasing, decreasing or constant practice performance in the context of distributed practice with regard to retention.

Concerning the learner characteristics, the explorative analyses in our study provided no hints for moderation effects. However, the results have to be interpreted cautiously given the relatively small sample size and the explorative character of the analyses. Thus, future studies concerned with distributive practice in particular—and with desirable difficulties in learning in general (Bjork, 1994)—should consider such individual aspects before recommending particular learning strategies to all learners in the same way.

To conclude, the effect of distributed practice on the retention of procedural skills requires further clarification before distributed practice can be recommended as an effective learning strategy in mathematics. Moreover, if no such effect exists, theoretical approaches are needed to explaining why the effect is existent for declarative knowledge but not for procedural knowledge. First considerations have been presented here (i.e., complexity; inferences between procedural and declarative memory; discrete motor procedures), but more research is required to check these assumptions.

## Data Availability Statement

The dataset for this study and the list of variables can be found in the OSF (https://osf.io/f6jqp/ and https://osf.io/b4fz7).

## Ethics Statement

Ethical review and approval was not required for the study on human participants in accordance with the local legislation and institutional requirements. The patients/participants provided their written informed consent to participate in this study.

## Author Contributions

Both authors contributed in equal parts to this manuscript. ME provided the general idea of this research, supervised its realization, and wrote large parts of the manuscript. KB specified the design of this study, collected and analyzed the data, and wrote the results section. Both authors revised the previous versions of this manuscript.

## Conflict of Interest

The authors declare that the research was conducted in the absence of any commercial or financial relationships that could be construed as a potential conflict of interest.

## Footnotes

1 This study was pre-registered: https://osf.io/y79s5/. It was carried out in accordance with the Ethical Principles of Psychologists and Code of Conduct (American Psychological Association, 2017). The local ethics committee does not claim a general check of studies in psychology.

2 According to GPower (Faul et al., 2009; version 3.1), a sample size of *N* = 138 would be required, assuming a mean effect size of *f*^2^ = 0.15 and a power of 1–β = 0.95, when the data were analyzed by a multiple linear regression, including five predictors (i.e., RI35 compared to RI7, ISI1 compared to ISI0, ISI11 compared to ISI1, and plus the two interaction terms).

3 For the sake of using time efficiently within the three sessions, the working memory of students in the massed condition was tested in the first session, while it was tested in the second session with students in the distributed condition – in both cases after the practice trials. Furthermore, concentration ability was assessed after the test in the third session in both learning conditions.

## Appendix A

### Practice and Test Tasks

In each task, participants were asked to calculate in how many unique ways the presented letters can be arranged. Correct solutions are indicated in parentheses.

#### Practice Set 1

abbc (12); abbbcc (60); aaaabc (30); and aaccccc (21).

#### Practice Set 2

accc (4); aaaacccc (70); aabbcc (90); and aacccc (15).

#### Test

aacc (6); aaacc (10); abbbbbbc (56); aaaaabc (42); and aacccccc (28).

## Appendix B

### Results of the Conditional Inference Tree Analyses

Regarding the test performance after *one week*, the performance in the first practice set (max. 5 points) yielded an effect, with poor performers (i.e., ≤2 points; *n* = 73) demonstrating a poorer test performance than medium performers (i.e., 3 points; *n* = 20), *p* < 0.001, who performed worse than good performers (i.e., >3 points, *n* = 27), *p* = 0.033. Working memory, assessed by the digit span, had an effect on the test performance after one week, with learners with a shorter digit span (i.e., ≤7; *n* = 89) achieving lower test scores than learners with a longer digit span (i.e., >7; *n* = 31), *p* = 0.032. Work avoidance had an effect, too, with learners with a higher work avoidance (i.e., >2.75; *n* = 23) yielding lower test scores than learners with a lower work avoidance, i.e., ≤2.75; *n* = 97), *p* = 0.044. Working memory, assessed by the Corsi block, sustained attention, effort motivation, and concentration yielded no effects and no interactions between the individual learners’ variables and the learning conditions (i.e., ISI) were revealed.

Regarding the test performance after *five weeks*, there was an effect of the performance in the first practice set, too. Poor performers (i.e., ≤2 points; *n* = 73) demonstrated a poorer test performance than better performers (i.e., >2 points; *n* = 40), *p* < 0.001. In addition, there was an effect of working memory, as assessed by the backward digit span: Students with a shorter to normal digit span (i.e., ≤8; *n* = 106) showed a poorer performance in the final test than students with a large digit span (i.e., >8; *n* = 8), *p* = 0.004. None of the other variables yielded a main effect on the final test performance after 5 weeks and no interactions could be assumed with the learning conditions (i.e., ISI).
